# Intelligence

**Published:** 1829-12

**Authors:** 


### INTELLIGENCE.

### MONTHLY REPORT OF DISEASES.

WE have recently seen two instances of Herpes Zoster (shingles), which were attended with symptoms of great disturbance to the general health. In most cases this complaint is rather distressing to the patient, from the irritation it produces, than alarming from its constitutional effects. Our best authority upon cutaneous diseases, BATEMAN, whose work has lately been so much improved by Dr. THOMSON, appears inclined to doubt even its occasional severity. He has "never in any instance witnessed any untoward symptom;" nor has he known it "followed by much debility."

The cases which have at this moment directed our attention to the subject, were similar to several others we have seen in the course of the last few years, and most of them occurring in persons of an advanced age. For an uncertain period, (either a few days or only several hours previous to the appearance of the eruption,) the patients we refer to were attacked with very violent darting pains through the chest and epigastrium; great restlessness; mental and bodily depression; a low and almost imperceptible pulse, and cold clammy sweats; sometimes paroxysmal attacks of palpitation of the heart and dyspnœa. We have not observed the "constant derangement of the digestive organs" as one of the precursory symptoms, which it is stated to be by RENAULDIN and other writers. From the continuance of pain, irritation, &c. the functions of the stomach are usually disturbed during the progress of the complaint. The appearance of the eruption is not generally followed by a decided diminution of the severity of the constitutional symptoms. In some cases we have seen them aggravated at that period, and continue for many days with unabated violence, and followed by great debility.

Various means have been suggested to relieve the distressing itching and intense heat which in violent cases attends the eruption. These symptoms we have always found benefited by warm fomentations, assiduously applied. In one case, cold vinegar and water had been tried as a local application by the patient. No alteration of the eruption followed, but both the local and general symptoms were rendered much more violent. We are informed by an experienced friend, that the itching and heat attending the eruption are relieved by the application of a solution of lunar caustic; about two grains to an ounce of water. To abate the severe lancinating pains in the chest, opiates should be freely given.

Although the disease is not unfrequently severe, we are not aware it has ever proved fatal. The opinion that herpes zoster certainly destroys if the eruption entirely encircles the body, is admitted by all modern writers to be erroneous. It originated, we believe, with Pliny, who states "Qui zoster appellatur enecat si cinxerit." (Nat. Hist. lib. xxvi. cap. xi.)

We have been induced briefly to mention the above cases, as proofs that the disease is not always so mild or trifling as the practitioner might expect from the statements of systematic writers upon cutaneous diseases.

### THE SIAMESE BOYS.

THE curiosity of the public, and especially of the medical public, has been raised to the highest pitch by the appearance of these extraordinary youths in the metropolis. As our readers must have perused some of the many descriptions which have been given in the public papers of this remarkable freak of nature, we shall confine ourselves to a brief mention of the most striking circumstances appertaining to the phenomenon.

These boys are about eighteen years of age. They are attached to each other by a bond of union, extending from the cartilago ensiformis to the umbilicus. In this connecting medium there is no pulsation whatever perceptible. It has a firm fleshy feel, excepting at the upper part, where the structure, during the course of the last six years, has gradually become harder, and is now as firm as cartilage. In no part of this attaching substance is there any appearance of ossification. There is but *one* umbilicus, which is situated in the middle of the lower margin of the bond of union. The pulsation of the hearts of the two boys is synchronous. With the exception of the preternatural connexion we have described, both boys are naturally formed. They enjoy a perfect state of health, and have never suffered from any disease but smallpox, with which they are slightly marked. One, indeed, had recently an attack of toothach, and during his indisposition the other suffered somewhat from mental anxiety, but his bodily health remained unaffected. They have never taken medicine of any kind. Their alvine evacuations occur nearly at the same time. Their ordinary and most natural position is face to face, but they are able to stand side by side without any inconvenience, the arm of one being generally thrown round the neck or the waist of the other. Their movements, which are performed with perfect facility, and even some grace, have been correctly compared to the evolutions of persons in the act of waltzing. They occasionally speak to each other, but have never been known to keep up a continued conversation.

We cannot perceive the slightest reason to suppose that one mind presides over the two bodies. They are, no doubt, distinct and perfect individuals as far as regards their mental faculties, and in all probability the only variation which exists in their corporeal structure from a natural formation, is the accidental bond of union we have described. It is true that their movements are simultaneous, or nearly so, but this circumstance must be regarded as the effect of habit and necessity. It may be worth while to mention that, when engaged at a game of draughts with a friend, the one corrected a mistake which the other was about to commit.

They have a strong sense of moral decorum, and are averse to any particular examination of their persons before many witnesses. Upon being asked if they would be gratified by the society of females, they replied they would wait until their return to their native country, when they would have no objection to marry. They are remarkably cheerful in temper, and apparently quick in intellect. We observed that they examined with eager curiosity every novel object.

Upon a first and superficial inspection of the part by which they are united, their separation might be thought practicable and safe; but we believe there is but one opinion upon this subject among those who have given it a proper consideration. The attempt would be neither justifiable nor safe. Not justifiable, because the boys themselves are shocked at the idea of separation; and unsafe, because there is every reason to believe that the abdominal cavity must be laid open in the attempt. When one boy coughs, the uniting substance feels as if some part of the viscera were forced into it. In the event of the death of one, their cautious separation would become an act of necessity, although we think it more than probable that neither would long survive the shock of losing his mate.

It has been said that their intellectual powers are unequal, but it would require greater opportunities of observation than we have had to form any opinion upon this subject. In features they strongly resemble each other, although a difference is perceptible upon a close examination. They have never been known to have the least dispute or contention with each other. The story that has passed through many of the public journals of one wishing for a cold, while the other was determined upon a hot, bath, is unfounded. So far, then, they have presented a rare instance of undisturbed fraternal harmony, and whatever difference of opinion may arise between them in their future progress through life, still they must remain the—united brethren.

It is gratifying to perceive the very affectionate attention which these youths receive from Captain Coffin. We have permission to see them some evening when they have retired from the exhibition of the day, and if any additional circumstances should strike us as worthy of mention, we shall communicate them to our readers.

### MONTHLY LIST OF MEDICAL BOOKS.

### METEOROLOGICAL JOURNAL,

By Messrs. HARRIS and Co. Mathematical Instrument Makers, 50, High Holborn.

| October | Moon | Rain gauge. | Thermom. 9 a.m. | Thermom. max. | Thermom. min. | Barometer. 9 A.M. | Barometer. 10P.M. | De Luc's Hygrom. 9 A.M. | De Luc's Hygrom. 10P.M. | Winds. 9 A.M. | Winds. 10P.M. | Atmospheric Variations. 9 a.m. | Atmospheric Variations. 2 p.m. | Atmospheric Variations. 10 p.m. |
|---|---|---|---|---|---|---|---|---|---|---|---|---|---|---|
| 20 | | .15 | 58 | 60 | 49 | 29.72 | 29.66 | 65 | 60 | SW | WSW | Cloudy | Fine | Show'ry |
| 21 | | | 55 | 58 | 49 | .64 | .53 | 61 | 61 | SSW | SSW | Fine | Fine | Fine |
| 22 | | | 52 | 54 | 44 | .54 | .72 | 61 | 59 | WSW | NNW | Cloudy | Fine | Cloudy |
| 23 | | | 49 | 50 | 41 | .82 | .85 | 59 | 60 | NNW | N | Foggy | Fine | Fine |
| 24 | | | 49 | 51 | 40 | .83 | .82 | 60 | 60 | NNE | NE | Fine | — | — |
| 25 | | | 45 | 51 | 43 | 30.01 | 30.06 | 62 | 62 | WSW | SW | Foggy | Fine | Fine |
| 26 | | | 47 | 50 | 42 | .17 | .20 | 65 | 65 | N | NNE | Cloudy | Fine | Fine |
| 27 | ● | | 48 | 51 | 41 | .25 | .19 | 63 | 61 | NNE | NNE | Foggy | Fine | Cloudy |
| 28 | | | 46 | 50 | 40 | .16 | .21 | 59 | 59 | NE | NNE | Fine | — | Fine |
| 29 | | | 44 | 52 | 41 | .21 | .14 | 60 | 61 | NNE | N | — | — | — |
| 30 | | | 46 | 51 | 40 | 29.90 | 29.78 | 61 | 58 | W | W | Cloudy | Fine | Cloudy |
| 31 | | | 44 | 51 | 39 | .80 | .93 | 58 | 55 | N | N | — | — | — |
| Nov. | | | | | | | | | | | | | | |
| 1 | | | 40 | 42 | 36 | .97 | 30.02 | 55 | 57 | WNW | WNW | Fine | — | Fine |
| 2 | | | 40 | 47 | 40 | 30.06 | .10 | 57 | 59 | WNW | WNW | Foggy | Cloudy | — |
| 3 | | | 44 | 47 | 45 | .00 | 29.99 | 59 | 62 | WNW | WNW | — | — | Cloudy |
| 4 | ☽ | | 49 | 51 | 40 | 29.79 | .50 | 65 | 65 | WSW | SW | Rain | Rain | Rain |
| 5 | | .50 | 43 | 50 | 44 | .85 | .91 | 65 | 65 | WNW | W | Cloudy | Fine | Fine |
| 6 | | | 47 | 51 | 43 | .84 | .75 | 65 | 63 | W | W | Fine | Fine | Cloudy |
| 7 | | | 45 | 52 | 36 | .78 | .80 | 63 | 60 | W | W | — | — | Fine |
| 8 | | | 41 | 47 | 40 | .83 | .88 | 61 | 61 | WNW | WNW | — | — | — |
| 9 | | | 44 | 52 | 45 | .91 | .97 | 61 | 62 | WNW | NW | — | — | — |
| 10 | | | 50 | 50 | 42 | .88 | .66 | 63 | 63 | WSW | SW | Fine | Cloudy | Rain |
| 11 | ○ | .13 | 47 | 55 | 47 | .82 | .82 | 63 | 65 | WNW | WSW | Foggy | Cloudy | Cloudy |
| 12 | | | 52 | 55 | 45 | .80 | .74 | 66 | 70 | WSW | W | Rain | Show'ry | Rain |
| 13 | | .17 | 53 | 58 | 40 | .92 | 30.05 | 68 | 65 | E | E | Cloudy | Fine | Fine |
| 14 | | | 42 | 46 | 38 | 30.01 | 29.97 | 65 | 65 | SE | SSW | Cloudy | Fine | Rain |
| 15 | | | 46 | 52 | 35 | 29.98 | 30.00 | 63 | 63 | SW | N | Show'ry | Fine | Show'ry |
| 16 | | .20 | 40 | 42 | 31 | 30.00 | .19 | 63 | 60 | N | NNE | Fine | — | Fine |
| 17 | | | 36 | 42 | 35 | .24 | .18 | 60 | 64 | NNE | N | Fine | Fine | Foggy |
| 18 | ☾ | | 39 | 41 | 32 | .24 | .28 | 64 | 65 | N | N | Foggy | Fine | Foggy |
| 19 | | | 33 | 36 | 29 | .30 | .27 | 65 | 65 | N | N | Th. Fog | Fog | Fog. |

The quantity of Rain fallen in the month of October, was 1 inch and 49-100ths.

